# Smartphone addiction, sleep quality, depression, anxiety, and stress among medical students

**DOI:** 10.3389/fpubh.2023.1252371

**Published:** 2023-09-06

**Authors:** Aleksandra Nikolic, Bojana Bukurov, Ilija Kocic, Milica Vukovic, Nikola Ladjevic, Miljana Vrhovac, Zorana Pavlović, Jovan Grujicic, Darija Kisic, Sandra Sipetic

**Affiliations:** ^1^Faculty of Medicine, Institute of Epidemiology, University of Belgrade, Belgrade, Serbia; ^2^Clinic of Otorhinolaryngology and Maxillofacial Surgery, Faculty of Medicine, University Clinical Center of Serbia, University of Belgrade, Belgrade, Serbia; ^3^Covid Hospital Krusevac, University Clinical Center of Niš, Niš, Serbia; ^4^Faculty of Medicine, Institute of Social Medicine, University of Belgrade, Belgrade, Serbia; ^5^Urology Hospital, University Clinical Centre of Serbia, Belgrade, Serbia; ^6^Faculty of Medicine, University of Belgrade, Belgrade, Serbia; ^7^Clinic for Psychiatry, Faculty of Medicine, University Clinical Center of Serbia, University of Belgrade, Belgrade, Serbia; ^8^University of Arkansans for Medical Science, Little Rock, AR, United States

**Keywords:** smartphone addiction, sleep quality, depression, anxiety, stress

## Abstract

**Introduction:**

Studies consistently link excessive smartphone use to poor sleep quality, depression, anxiety, and stress. This study specifically aimed to investigate these associations among medical students in Belgrade and Nis (Central Serbia).

**Materials and methods:**

The cross-sectional study included a sample of 761 students, who were selected from both the Faculties of Medicine at the University of Belgrade and the University of Nis. Questionnaires, including the International Physical Activity Questionnaire – Short Form (IPAQ-SF), Smartphone Addiction Scale – Short Version (SAS-SV), the Pittsburgh Sleep Quality Index (PSQI), and the Depression, Anxiety, and Stress Scale – 21 items (DASS-21), were completed by the participants. Statistical analysis techniques, such as the Chi-square test, student’s *t*-test, and logistic regression, were employed to examine the relationship between smartphone addiction, physical activity, sleep quality, depression, anxiety, and stress.

**Results:**

The findings indicated a prevalence of smartphone addiction among medical students at 21.7%, with rates of 22.9% among males and 21.1% among females. Females exhibited significantly higher scores on the SAS-SV scale compared to males (*p* = 0.032). Univariate logistic regression analysis revealed significant associations between smartphone addiction and spending over 4 h daily on smartphones (OR = 2.39; *p* < 0.001), poor sleep quality (OR = 1.65; *p* = 0,005), as well as elevated levels of stress (OR = 1.75; *p* = 0.003), anxiety (OR = 2.04; *p* < 0.001), and depression (OR = 2.29; *p* < 0.001). Multivariate regression analysis identified spending more than 4 h daily on smartphones (OR = 2.39; *p* < 0.001) and increased levels of depression (OR = 2.51; *p* < 0.001) as independent significant factors associated with smartphone addiction.

**Conclusion:**

This study sheds light on the prevalence of smartphone addiction among medical students, with spending excessive time on smartphones and higher levels of depression standing out as significant factors. Future research should delve into the underlying mechanisms and causal relationships between smartphone addiction and these psychosocial factors. Understanding these connections will aid in developing effective interventions and strategies to tackle this growing public health concern.

## 1. Introduction

A smartphone is a portable mobile phone with computer-like capabilities (1). Unlike traditional mobile phones that were primarily used for calls and SMS messages, smartphones now offer a wide array of functionalities, such as email, music, camera, web browsing, gaming, and a multitude of applications. Currently, around 6.6 billion people worldwide use smartphones, and this number is projected to reach 7.8 billion by 2028 (2).

The increase in smartphone usage has led to a surge in research investigating excessive and problematic smartphone use. Many studies indicate that a significant number of individuals engage in excessive phone use, leading to disruptions in their daily routines, safety, and general well-being (3–6). Furthermore, prolonged and excessive smartphone usage has been associated with negative effects on mental health and behavior (7–9).

Problematic smartphone use is often conceptualized as a behavioral addiction, exhibiting similarities to non-chemical addictions such as gambling addiction. This condition is characterized by a diminished ability to regulate phone use, accompanied by addiction-related symptoms such as tolerance, withdrawal, preoccupation, unsafe or prohibited phone usage, and adverse functional consequences (10). Despite the abundance of research highlighting the addictive characteristics of smartphones, smartphone addiction has not yet received official recognition in the Diagnostic and Statistical Manual of Mental Disorders (DSM) published by the American Psychiatric Association (11), nor in the International Classification of Diseases (ICD) (12). Nonetheless, although it lacks formal diagnostic status, criteria for smartphone addiction have been suggested (13). The criteria for smartphone addiction can be categorized into three sections: Part A, comprising six symptom criteria that delineate specific symptoms of smartphone addiction; Part B, focusing on functional impairments that arise from excessive smartphone use; and Part C, consisting of exclusion criteria that rule out manic episodes or obsessive-compulsive disorder (13). Research findings revealed significant overlaps between the characteristics of smartphone addiction and substance-related or behavioral addictive disorders (13). Additionally, Horvath et al. (14) provided evidence of specific structural and functional correlates associated with behavioral addictions in individuals who met the psychometric criteria for smartphone addiction.

Contrary to the prevailing notion of smartphone addiction, some studies have raised questions regarding its conceptualization (15, 16). Some authors argue that the debates surrounding the understanding and acceptance of technological and behavioral addictions are largely influenced by terminology. They note that while smartphone addiction may not have the same severity and health consequences as substance addiction, there is no alternative widely accepted term to describe the lack of self-control, attachment, overuse, and adverse outcomes associated with this behavior (15). Consequently, “addiction” has become an accepted umbrella term despite potential misrepresentation of the disorder’s severity and the resulting implications for research and treatment efforts. The concept of “smartphone addiction” continues to be a subject of extensive debate in scientific literature, with ongoing controversies surrounding its terminology. Nevertheless, “smartphone addiction” has gained widespread acceptance and usage in the field’s literature. Therefore, in alignment with existing conventions, this study employs the term “smartphone addiction” to characterize the excessive use of smartphones that adversely affects daily functioning.

Multiple studies have provided evidence of a positive correlation between smartphone addiction and stress, anxiety, and depression (8, 17), as well as associations with poor sleep quality, fatigue, difficulties in falling asleep, and shorter sleep duration (17–20). Moreover, in clinical settings, excessive smartphone use can serve as a significant distraction and lead to potential negligence and harm to patient health (21). To explore the relationship between “smartphone addiction” and various factors including physical activity, depression, anxiety, stress, and sleep quality, a cross-sectional study was conducted on a representative sample of students from the Faculty of Medicine in Belgrade and the Faculty of Medicine in Nis.

## 2. Materials and methods

This cross-sectional study was conducted between 1st and 31st December 2018 among students of medical faculties in Belgrade and Nis, Serbia. The study was reviewed and approved by the Ethics Committee of the Faculty of Medicine, University of Belgrade (No. 2650/XII-1). The Dean of the Medical Faculty at the University of Nis gave signed permission to conduct research among medical students in Nis. Students were offered the opportunity to voluntarily complete the questionnaire at the beginning of their classes. Informed consent was obtained from all participants before completing the questionnaire survey.

### 2.1. Participants

The study was conducted on a representative sample of medical students from the Faculty of Medicine University of Belgrade (MFUB) and the Faculty of Medicine University of Nis (MFUN), Republic of Serbia. Multi-stage sampling was used. The required sample size for MFUB student population was computed as 291 using Epi Info 7 (version 7.2.5.0) [population size: 3,055 medical students; expected frequency: 29.8% (22); acceptable margin of error: 5%; design effect: 1]. The required sample size for MFUN student population of 253 was computed using Epi Info 7 (version 7.2.5.0) [population size: 1198 medical students; expected frequency: 29.8% (22); acceptable margin of error: 5%; design effect: 1]. To address the possibility of incomplete questionnaires, a larger number of students was included in the study. A total of 761 students completed the questionnaire (383 students (50.3%) from the MFUB and 378 (49.7%) students from the MFUN). Students from 1st to 6th year of study were evenly distributed in the sample.

### 2.2. Instruments

Respondents completed various self-report questionnaires. A specifically developed questionnaire was utilized to collect sociodemographic characteristics of the participants, including gender, age, year of study, place of residence during studies (with parents/apartment/room/dorm/other), socioeconomic status (good/medium/poor), grade point average (GPA) (for students in their 2nd to 6th year of study; with the minimum grade being 6 and the maximum being 10), body mass (in kilograms), body height (in meters), smoking status (smokers/non-smokers), alcohol consumption (yes/no), and energy drinks consumption (yes/no). Additionally, participants were asked about their daily time spent on smartphones. In the logistic regression step (Tables 1, 2), the numerical variables age, year of study, GPA, and “time spent on smartphones” were dichotomized to identify predictors of smartphone addiction.

**Table 1:** Factors associated with smartphone addiction among students of medical faculties: results of univariate logistic regression analysis.

	Not addicted *N* = 596 *N* (%)	Addicted *N* = 165 *N* (%)	OR (95%CI)	*p*-value
Sex, female	411 (69.0)	110 (66.7)	0.90 (0.62–1.30)	0.575
Age ≤ 21 years	284 (47.7)	74 (44.8)	0.89 (0.63–1.26)	0.523
Year of study ≤3	300 (50.3)	81 (49.1)	0.95 (0.67–1.34)	0.777
GPA ≥ 8.8	246 (51.2)	63 (45.7)	0.80 (0.55–1.17)	0.247
Residence during studies – With parents	244 (40.9)	77 (46.7)	1.26 (0.89–1.78)	0.188
Socio-economic status – Good	356 (59.7)	97 (58.8)	0.96 (0.67–1.37)	0.827
Parents’ marital status – Married	485 (82.3)	139 (86.3)	1.36 (0.82–2.23)	0.231
Smoking	119 (20.0)	32 (19.4)	0.96 (0.62–1.49)	0.870
Alcohol consumption	391 (65.6)	120 (72.7)	1.40 (0.95–2.05)	0.086
Energy drink consumption	227 (38.2)	69 (41.8)	1.16 (0.82–1.66)	0.393
**BMI categories**^†^				
Underweight	55 (9.3)	17 (10.4)	1 (Ref.)	
Normal weight	445 (74.9)	122 (74.4)	0.89 (0.49–1.58)	0.685
Overweight	84 (14.1)	23 (14.0)	0.89 (0.43–1.81)	0.739
Obesity	10 (1.7)	2 (1.2)	0.65 (0.13–3.25)	0.597
**Physical activity**				
Low	120 (20.1)	31 (18.8)	1 (Ref.)	
Moderate	291 (48.8)	78 (47.3)	1.04 (0.65–1.66)	0.650
High	185 (31.0)	56 (33.9)	1.17 (0.71–1.92)	0.714

X̄, Mean; SD, Standard Deviation; OR, Odds Ratio; 95%CI, 95% Confidence Interval; GPA, Grade Point Average—average grade for students in their 2^nd^ to 6^th^ year of study; *p*-value for ULRA; BMI, Body Mass Index; ^†^The body height or weight data of 3 students was not provided, rendering the calculation of their BMI unfeasible.

**Table 2:** Factors associated with smartphone addiction among students of medical faculties: results of univariate^*^ and multivariate^**^ logistic regression analysis.

Characteristics	Not addicted *N* = 601	Addicted *N* = 164	OR (95%CI)^*^	*p* value^*^	OR (95%CI)^**^	*p* value^**^
Time spent on smartphone >4 h/day	181 (30.4)	81 (49.1)	2.21 (1.55–3.13)	**<0.001**	2.39 (1.66–3.43)	**<0.001**
**Sleep quality**						
Good	376 (63.1)	84 (50.9)	1.65 (1.16–2.33)	**0.005**		
Poor	220 (36.9)	81 (49.1)				
**Stress**						
Normal	449 (75.3)	105 (63.6)	1 (Ref.)			
Mild	69 (11.6)	16 (9.7)	0.99 (0.55–1.78)	0.977		
Moderate	53 (8.9)	33 (20.0)	2.66 (1.64–4.32)	**<0.001**		
Severe	19 (3.2)	9 (5.5)	2.03 (0.89–4.61)	0.092		
Extremely severe	6 (1.0)	2 (1.2)	1.42 (0.28–7.16)	0.667		
Increased stress^†^	147 (24.7)	60 (36.4)	1.75 (1.21–2.52)	**0.003**		
**Anxiety**						
Normal	408 (68.5)	85 (51.5)	1 (Ref.)			
Mild	47 (7.9)	17 (10.3)	1.74 (0.95–3.17)	0.072		
Moderate	83 (13.9)	29 (17.6)	1.68 (1.03–2.72)	**0.036**		
Severe	30 (5.0)	10 (6.1)	1.60 (0.75–3.40)	0.221		
Extremely severe	28 (4.7)	24 (14.5)	4.11 (2.27–7.45)	**<0.001**		
Increased anxiety^†^	188 (31.5)	80 (48.5)	2.04 (1.44–2.90)	**<0.001**		
**Depression**						
Normal	451 (75.7)	95 (57.6)	1 (Ref.)			
Mild	59 (9.9)	26 (15.8)	2.09 (1.25–3.49)	**0.005**		
Moderate	48 (8.1)	26 (15.8)	2.57 (1.52–4.35)	**<0.001**		
Severe	22 (3.7)	9 (5.5)	1.94 (0.86–4.35)	0.107		
Extremely severe	16 (2.7)	9 (5.5)	2.67 (1.14–6.22)	**0.023**		
Increased depression^†^	145 (24.3)	70 (42.4)	2.29 (1.59–3.29)	**<0.001**	2.51 (1.73–3.63)	**<0.001**

OR, Odds Ratio; 95% CI, 95% Confidence Interval; ^*^p value for ULRA; ^**^p value for MLRA. Bold values indicate statistical significance. ^†^ dichotomous variables (normal vs. increased).

The level of smartphone addiction was assessed using the short version of the Smartphone Addiction Scale (SAS-SV) (23). The items on the SAS-SV were carefully selected from the original Smartphone Addiction Scale (SAS), which consisted of 33 items, based on their validity (24). The correlation between the SAS-SV and SAS was found to be 0.96. The SAS-SV is a validated scale comprising 10 items that participants rate on a 6-point Likert scale (ranging from 1 = strongly disagree to 6 = strongly agree). The total score on this scale can range from 10 to 60, with higher scores indicating a greater degree of smartphone addiction. The original SAS-SV demonstrated content and concurrent validity, as well as internal consistency (Cronbach’s alpha of 0.91). The items on the scale encompass various aspects of addiction such as disruption in daily life, withdrawal symptoms, relationships centered around cyberspace, excessive use, and tolerance. The SAS-SV possesses the advantage of being able to identify a potentially high-risk group for smartphone addiction, both within educational settings and the broader community. For males, a cut-off value of 31 is considered indicative of addiction (with a sensitivity of 0.867 and specificity of 0.893), while for females, the corresponding cut-off value is 33 (with a sensitivity of 0.875 and specificity of 0.886). In this study, a translated and culturally adapted Serbian version was used, which proved to be a valid and reliable instrument for assessing smartphone addiction (25).

Depression, anxiety, and stress levels were evaluated utilizing the DASS-21 scale (Depression Anxiety and Stress Scale - DASS). The DASS-21 comprises three subscales designed for self-assessment of negative emotional states associated with depression, anxiety, and stress (26). In essence, the Depression subscale predominantly captures low positive affect, the Anxiety subscale focuses on physiological arousal, and the Stress subscale evaluates nonspecific negative affect (26, 27). Respondents indicate the extent to which they experienced each listed condition in the previous week using a four-point response scale. The responses for each subscale are then summed and multiplied by two to ensure comparability with results obtained from the DASS-42 scale. Based on the scores, the subscales are categorized as follows: normal, mild, moderate, severe, extremely severe (28). To facilitate multivariate logistic regression analysis, the variables were classified into the following two distinct categories: normal and increased. The Serbian translation of the DASS-21 scale underwent psychometric testing on a sample of students (1,374 students) in Novi Sad (27). The results demonstrated that the Serbian version of DASS-21 is a reliable and valid measure of unpleasant emotional states.

Sleep quality was assessed using the Pittsburgh Sleep Quality Index (PSQI), a questionnaire designed to measure subjective sleep quality over a one-month period (29). The PSQI consists of 19 self-rated questions, as well as five questions answered by a bed partner. The additional questions answered by another person, typically a partner or roommate, provide clinical information but are not included in the scoring process. The 19 items of the PSQI are divided into seven components, namely subjective sleep quality, sleep latency, sleep duration, sleep efficiency, sleep disturbances, use of sleep medication, and daytime dysfunction. Each component is assigned a score, and the scores for the seven components are summed to obtain the global PSQI score. The global score ranges from 0 to 21, with higher scores indicating poorer sleep quality. The Serbian translation of the PSQI questionnaire has been validated and can be utilized as a reliable screening tool for assessing sleep quality in diverse populations (30). A cutoff score of 5 is commonly used to categorize respondents into two groups: those with good sleep quality (PSQI <5) and those with poor sleep quality (PSQI ≥5) (30).

The International Physical Activity Questionnaire-Short Form (IPAQ-SF), a reliable and validated tool, was employed to evaluate physical activity levels over the past week (31). The IPAQ-SF captures four levels of physical activity intensity: vigorous-intensity physical activity, which encompasses activities like heavy lifting, intense aerobic exercises, and using a bike or treadmill; moderate-intensity physical activity, including tasks such as carrying light loads, cycling at a regular pace, and engaging in yard work; walking time; and average sitting time on weekdays, including sedentary work. Furthermore, the IPAQ-SF provides information on the level of physical activity in terms of energy expenditure measured in metabolic equivalent (MET)-minutes per week (32). MET-min/week coefficients used for each type of physical activity were: 3.3 for walking, 4.0 for moderate physical activity, and 8.0 for vigorous physical activity. The total weekly energy expenditure, known as total physical activity, was determined by summing the MET-min/week values for walking, moderate physical activity, and vigorous physical activity. Based on the total MET score, respondents were categorized into three groups: high, moderate, and low physical activity levels. Respondents with a weekly energy expenditure of less than 600 MET-minutes were classified as having low physical activity, those with 600–3,000 MET-minutes fell into the moderate physical activity category, and individuals with more than 3,000 MET-minutes were categorized as engaging in high-intensity physical activity. A spreadsheet programmed with specific criteria was utilized to automatically calculate the scores (33).

The body mass index (BMI) was employed as a measure to evaluate the nutritional status of the participants. Participants provided their height and weight, and the BMI was calculated based on these values. All participants were classified into four groups based on their BMI: underweight (BMI < 18 kg/m^2^), normal weight (BMI = 18–24.9 kg/m^2^), overweight (BMI = 25.0–29.9 kg/m^2^), and obesity (BMI ≥ 30 kg/m^2^) (34).

### 2.3. Statistical analysis

The statistical analysis of the data was performed using the SPSS 23.0 program (SPSS Inc., Chicago, IL, United States) for data processing. Descriptive statistics methods were employed, including the calculation of mean values, standard deviations, medians, minimum and maximum values. For comparing two groups of subjects, either a parametric test (independent samples *t*-test) or a non-parametric test (χ^2^ test) was applied. More specifically, the independent samples *t*-test was used to analyze numerical variables, while the Chi-square test was applied to investigate categorical variables. The observed differences were considered statistically significant if the *p*-value was less than 0.05. To identify predictors of smartphone addiction, both univariate logistic regression analyses (ULRA) and multivariate logistic regression analyses (MLRA) were conducted. The dependent variable in these analyses was smartphone addiction, assessed based on the recommended cut-off values of 31 for males and 33 for females. Independent variables in multivariate analysis included all variables that exhibited significance in the univariate analysis at a significance level of less than 0.05.

## 3. Results

The study included 761 students (Table 3). The gender distribution consisted of 68.5% males and 31.5% females. The sample was evenly distributed among students from the 1st to the 6th year of study, with each year comprising 124–130 students. On average, the age of the students was 21.81 (±2.15) years. The students’ average grade point average (GPA) was calculated to be 8.75 (±0.72). Around 42% of the students resided with their parents during their studies. Socio-economic status indicated that approximately 60% of the students had a good socioeconomic standing, 38% had a medium standing, and 2.5% had a poor standing. Approximately 83% of students in both faculties had parents who were married.

**Table 3:** Demographic characteristics of students from medical faculties.

Demographic characteristics	Total (*N* = 761)
**Sex, *N* (%)**	
Male	240 (31.5)
Female	521 (68.5)
Age, years (X̄±SD)	21.81 ± 2.15
**Year of study, *N* (%)**	
1	128 (16.8)
2	128 (16.8)
3	125 (16.4)
4	130 (17.1)
5	126 (16.6)
6	124 (16.3)
GPA, (X̄±SD)	8.75 ± 0.72
**Residence during studies, *N* (%)**	
With parents	321 (42.2)
Apartment/room/dorm/other	440 (57.8)
**Socio-economic status, *N* (%)**	
Good	451 (59.3)
Medium	291 (38.2)
Poor	19 (2.5)
Married parents, *N* (%)	632 (83.0)

X̄, Mean; SD, Standard Deviation; GPA, Grade Point Average—average grade for students in their 2^nd^ to 6^th^ year of study.

The mean value of the Smartphone Addiction Scale - Short Version (SAS-SV) score was found to be 24.69 (±9.14) (Table 4). The prevalence of smartphone addiction among the participants was determined to be 21.7%. Notably, females exhibited significantly higher scores on the SAS-SV scale compared to males. However, when considering the prevalence of smartphone addiction, no significant difference was observed between males and females.

**Table 4:** Smartphone addiction prevalence among males and females in medical faculties.

	Males (*N* = 240)	Females (*N* = 521)	Total (*N* = 761)	*p*-value
**SAS-SV score**				
X̄±SD	23.65 ± 9.00	25.17 ± 9.17	24.69 ± 9.14	0.032^*^
Median (min-max)	22 (10–58)	25 (10–58)	24 (10–58)	
**Smartphone addiction**				
Addicted	55 (22.9)	110 (21.1)	165 (21.7)	0.575^**^
Not addicted	185 (77.1)	411 (78.9)	596 (78.3)	

SAS-SV, Smartphone Addiction Scale – Short Version; X̄, Mean; SD, Standard Deviation; ^*^p value for Student’s *t*-test; ^**^p value for χ^2^ test. Bold values indicate statistical significance.

The findings of the univariate analysis examining factors associated with smartphone addiction among students are presented in Table 1. Variables including sex, age, year of study, GPA, housing during studies, socio-economic status, parents’ marital status, smoking, alcohol consumption, energy drink consumption, nutritional status and physical activity, were not found to be significantly associated with smartphone addiction.

Based on the findings from the univariate logistic regression analysis, it was observed that spending more than 4 h daily on smartphones, poor sleep quality, elevated levels of stress, anxiety, and depression were significantly associated with smartphone addiction (Table 2). Multivariate logistic regression models were utilized to identify the factors independently associated with smartphone addiction. The independent variables included in the multivariate analysis were those that showed significance in the univariate analysis at a level of *p* < 0.05, namely: time spent on the smartphone (>4 h per day), sleep quality, stress (dichotomous), anxiety (dichotomous), and depression (dichotomous). According to the final model, the significant factors independently associated with smartphone addiction were spending more than 4 h daily on smartphones (OR = 2.39; *p* < 0.001) and increased levels of depression (OR = 2.51; *p* < 0.001).

## 4. Discussion

In our study, the prevalence of smartphone addiction among medical students was determined to be 21.7%. According to the SAS-SV scale, smartphone addiction rates were estimated to be 12% among Japanese students (35), 16.9% in Switzerland (36), 26.9% in Romania (37), 29.8% in China (22), 33.1% in Brazil (38), and 35.9% in Thailand (35). The highest prevalence of addiction was observed in Saudi Arabia, reaching 71.9% (39), followed by India at 73%, and Iraq at 78.3% (40). Variations in the prevalence of smartphone addiction among students across countries may stem from differing social and cultural environments and disparities in the development and accessibility of information and communication technologies. Additionally, the choice of threshold value can influence reported prevalence, emphasizing the need for further studies to establish appropriate thresholds in diverse populations and age groups.

When analyzing the scores obtained from the SAS-SV scale, it was observed in our study that women had significantly higher scores than men. The higher scores achieved by women on the SAS-SV scale justified the recommended higher cutoff value (in order to be considered indicative of addiction) in the original study (23). However, in our study, no significant difference was observed between males and females when considering the prevalence of smartphone addiction. This finding aligns with the study conducted by Chen et al. (22), which also utilized the SAS-SV scale to assess smartphone addiction among medical students in China, revealing no significant difference in addiction prevalence between men (30.3%) and women (29.3%). In contrast, some studies have reported a higher prevalence of “smartphone addiction” in women (39). A review by Yu and Sussman (10) found that gender was a significant predictor in 19 studies (18% of the total studies reviewed), indicating that women had a higher risk of being “smartphone addicted.” Additionally, 16 studies demonstrated that women were more inclined to be addicted to social media, while men exhibited a greater likelihood of game addiction (10).

In our study, there was no difference in the prevalence of smartphone addiction across different age groups. Several studies have consistently demonstrated a higher prevalence of addiction among younger individuals (36). Notably, a substantial number of studies have focused on addiction in adolescents and young adults (19, 36). Within a comprehensive review encompassing 108 studies, a remarkable 78% of them specifically examined adolescents and young adults, consistently reporting higher prevalence rates of smartphone addiction among this age group compared to older adults (10). One crucial aspect contributing to the susceptibility of younger individuals to smartphone addiction is their developmental stage. Adolescence and young adulthood mark significant periods of identity formation and social interaction. During this time, young people are more likely to seek social validation, peer acceptance, and a sense of belonging. Smartphones offer a means to fulfill these needs by providing constant connectivity to social networks and online communities. The desire to stay connected and “fear of missing out” (FoMO) can drive excessive smartphone use as they strive to maintain social relationships and seek approval from their peers.

In our study, we observed no statistically significant distinction in the mean GPA scores between group of students classified as being addicted to smartphones and those classified as non-addicted. The findings from a study among college students revealed a significant association between increased cell phone usage and decreased academic achievement (6). Moreover, a comprehensive review encompassing 23 studies investigating the link between smartphone usage and academic performance in tertiary education institutions found that 18 studies (78.3% of the total) reported a significant negative correlation between smartphone use and academic achievement. The remaining five studies did not demonstrate a significant relationship (41). The research suggests that students predominantly perceive smartphones as recreational devices, primarily utilizing them for social networking, internet browsing, video consumption, and gaming (6, 42). Additionally, the constant need to remain connected and stay updated, driven by the “fear of missing out” (FoMO), can impair the focused attention required for achieving favorable academic results (43). Furthermore, due to a lack of academic motivation, students may experience boredom in class or during study periods, with smartphones and various applications offering a quick and tempting escape from this state of ennui (41, 44).

Based on the findings of our study, no significant differences were observed between students categorized as addicted and those categorized as non-addicted in terms of smoking behavior, alcohol consumption, and energy drink consumption. Conversely, numerous studies have reported a correlation between these behaviors and smartphone addiction. In a study conducted in China, it was demonstrated that current smokers and individuals who consume alcohol once a week or more frequently are at a higher risk of smartphone addiction (45). Both smartphone use and alcohol/cigarette consumption can serve as coping mechanisms for individuals dealing with stress, anxiety, or other emotional challenges. People may turn to their smartphones or alcohol/smoking as a way to escape from negative emotions or to seek temporary relief. In a study conducted among students in South Korea, factors such as female gender, alcohol consumption, internet addiction, and anxiety were identified as risk factors for smartphone addiction among students (46). This connection can be explained by a shared psychosocial process underlying excessive use of psychoactive substances and other addictions. Recognized risk factors associated with problematic phone use encompass various personality traits such as neuroticism, anxiety, depression, low self-esteem, low self-control, and impulsivity (16, 47, 48). These factors may create a vulnerability for individuals to engage in excessive smartphone use and substance abuse as a means of self-medication or self-soothing.

In our study, a significant association was observed between spending more than 4 h daily on smartphones and smartphone addiction. The variable of spending over 4 h per day on smartphones emerged as an independent factor significantly associated with smartphone addiction. Other researchers have also found similar results, indicating that increased frequency and duration of phone usage pose a risk for addiction (24, 39, 49). This suggests that students who are addicted face difficulties in controlling excessive phone use. In a study conducted among young individuals in Switzerland, significant independent predictors of smartphone addiction included longer phone usage, shorter time until the first phone use after awakening, and the predominance of social media usage as the primary activity on the phone (36).

In our study, no statistically significant difference was observed in terms of physical activity levels or nutritional status between students classified as addicted and those classified as non-addicted. Smartphone usage predominantly involves sedentary behavior (50). Extended periods spent gazing at smartphone and tablet screens have been linked to reduced engagement in physical activity, leading to higher body mass index, decreased physical activity levels, and various health issues such as visual impairments and musculoskeletal problems (39, 51, 52). Notably, students addicted to smartphones exhibit lower levels of physical activity compared to non-addicted students, a pattern observed in children as well (53). A mini-review investigating the impact of phone use on physical activity deficits revealed a negative association between smartphone usage and physical activity in 9 out of 14 studies (51). In a study conducted among Chinese students, students classified as being at a higher risk of smartphone addiction, exhibited lower muscle mass and higher fat tissue mass compared to those who exhibited moderate phone usage (54).

While excessive smartphone use can contribute to sedentary behaviors and reduced physical activity, it’s worth noting that smartphone use can also be leveraged to promote and improve engagement in physical activity. Smartphone applications designed for fitness tracking can monitor and record various physical activities, such as walking, running, cycling, or workout sessions. These apps can provide real-time feedback, track progress, set goals, and offer motivation, thereby encouraging individuals to engage in regular physical activity. Smartphone apps and social media platforms can provide a sense of community and social support for individuals pursuing physical activity goals. It’s important to note that while smartphones can be helpful tools for promoting physical activity, it’s crucial to find a balance and avoid excessive smartphone use that interferes with active and healthy lifestyles.

The present study revealed a significant association between smartphone addiction and various psychosocial factors, including poor sleep quality, elevated levels of stress, anxiety, and depression. These findings suggest that individuals who exhibit higher levels of smartphone addiction are more likely to experience compromised sleep patterns and increased psychological distress. But of all the factors listed, an increased level of depression was identified as an independent predictor of smartphone addiction. In a study conducted among college students in Turkey, a significant association was found between smartphone use and depression and anxiety, indicating that higher levels of phone use were correlated with increased symptoms of depression and anxiety (8). Similar findings were observed in a study conducted among students in Lebanon, where a positive association between smartphone addiction, anxiety, and depression was identified. Both depression and anxiety were found to be independent positive predictors of smartphone addiction (55). A systematic review conducted by Elhai et al. (56) demonstrated a consistent connection between excessive smartphone use, depression, anxiety, and stress. A meta-analysis investigating the relationship between problematic phone use and mental health, involving 41 studies with a total sample size of 41,871 children and youth, revealed that problematic smartphone use was associated with an increased risk of depression (OR = 3.17), anxiety (OR = 3.05), stress (OR = 1.86), and poor sleep quality (OR = 2.60) (57). These collective findings highlight the strong link between problematic smartphone use and mental health issues, emphasizing the need for further research and targeted interventions to address this growing concern.

In a broader context, the examination of the influence of the internet and technology reveals compelling evidence linking the co-occurrence of depression, anxiety, and stress to the development of technology addiction. For instance, individuals experiencing persistent stress may resort to internet-based video games as a coping strategy (58), whereas those suffering from depression may employ mobile phones as a means to alleviate negative emotions which leads to more time spent on phones (59). A follow-up study by Kang et al. (60) revealed that students experiencing depression and/or anxiety were more prone to engaging in addictive smartphone usage compared to individuals without mental health issues. In essence, the utilization of smartphones serves as a means to circumvent the engagement with unpleasant emotional content, thereby engendering a spectrum of potential consequences. Conversely, heightened engagement with technology can contribute to the onset of depression, anxiety, and stress. A cohort study involving students demonstrated that individuals classified as “high users,” specifically those who extensively utilize computers, social networks, and mobile phones, exhibited elevated levels of chronic stress, depression, and sleep disturbances after a one-year follow-up (61). Furthermore, a bidirectional association can be observed between excessive phone usage and psychological disorders, wherein problematic phone use can contribute to the development of psychological disorders, and conversely, pre-existing psychological disorders can lead to problematic phone use (62, 63). It can be postulated that a reciprocal relationship exists between phone addiction and mental disorders, forming a cyclical pattern with reinforcing effects.

In our study, the findings from the univariate logistic regression analysis revealed a significant association between poor sleep quality and smartphone addiction among students. In a previous study conducted among medical students, more than half of the students (54.7%) experienced poor sleep quality. Additionally, approximately one in every four students reported increased fatigue, and almost half of the students exhibited elevated levels of sleepiness (64). According to a study conducted by Chung et al. (19), individuals classified as “at-risk users” showed 2.3 times higher levels of daytime sleepiness compared to users of “low-risk.” Another study by Sohn et al. (65), which involved 1,043 students in London, found that 61.6% of the students had poor sleep quality. Among the participants, a significantly higher percentage of smartphone-addicted students, experienced poor sleep quality (68.7%) compared to non-addicted students (57.1%). This suggests that smartphone addiction is associated with poor sleep quality (OR = 1.41) (36). Furthermore, even after adjusting for daily screen time, the association between poor sleep quality and smartphone addiction remained significant, indicating that the link between these factors is not solely explained by the duration of phone use. A systematic review of existing literature by Hale et al. (66). indicate that the majority of studies consistently demonstrate a negative relationship between screen-based media consumption and sleep health. This association primarily manifests through delayed bedtimes and reduced overall sleep duration. The underlying mechanisms that contribute to these associations are likely as follows: (1) time displacement, where screen usage replaces time that could have been allocated to sleep and other activities; (2) psychological stimulation derived from the content of the media; and (3) the impact of the light emitted by electronic devices on circadian rhythm, sleep patterns, and wakefulness (67). Additionally, problematic smartphone use can contribute to the development of depression and/or anxiety, conditions that are known to be associated with sleep difficulties (8). Another way in which smartphones can have a negative impact on sleep quality is by causing nocturnal awakenings or interruptions during sleep. In today’s society, it is commonly assumed that almost everyone possesses a smartphone, and there is an expectation for individuals to be constantly accessible. Communication with others often persists even after retiring to bed (68).

It is important to consider the limitations of the study when interpreting the findings. The limitations of this study stem from its cross-sectional study design. Causal relationships cannot be established in cross-sectional studies, including the direction of the associations. Specifically, it cannot be concluded whether individuals with higher levels of stress, depression, and anxiety and poorer sleep quality are more prone to smartphone addiction, or if excessive smartphone use among individuals leads to increased levels of these negative emotional states and worsened sleep quality. Adequately designed longitudinal and clinical studies are necessary to investigate this association. Another limitation of the current study is that it relied on retrospective subjective reports. Therefore, it is recommended that future research include objectively recorded data on smartphone usage, body weight and height and physical activity. The study used the cut-off values of the SAS-SV score recommended by the questionnaire authors. Although the recommended cut-off values are widely used in research studies on different populations, it is advisable to assess the predictive value of this scale and the cut-off values in collaboration with psychiatrists and psychologists. However, despite the mentioned limitations, the SAS-SV scale is among the most commonly used and translated instruments for assessing smartphone addiction.

## 5. Conclusion

In our study, we found that the prevalence of smartphone addiction among medical students was determined to be 21.7%. The significant factors independently associated with smartphone addiction were spending more than 4 h daily on smartphones and increased levels of depression. It is crucial for future research to delve deeper into the underlying mechanisms and causal relationships between smartphone addiction and these psychosocial factors. This will enable the development of effective interventions and strategies to address this growing public health concern.

## Data availability statement

The raw data supporting the conclusions of this article will be made available by the authors, without undue reservation.

## Ethics statement

The studies involving humans were approved by Ethics Committee of the Faculty of Medicine, University of Belgrade (No. 2650/XII-1). The studies were conducted in accordance with the local legislation and institutional requirements. The participants provided their written informed consent to participate in this study.

## Author contributions

SS, AN, DK, and ZP contributed to the conception and design of the study. The database was organized by AN, MVu, NL, JG, MVr, and IK. AN, BB, and SS performed the statistical analysis. AN wrote the first draft of the manuscript. AN, MVu, NL, and JG wrote sections of the manuscript. SS, BB, and ZP reviewed and edited the manuscript. SS was responsible for project administration and funding acquisition. All authors contributed to the article and approved the submitted version.

## Funding

This research was funded by the Ministry of Education, Science and Technological Development of the Republic of Serbia (Project No. 200110).

## Conflict of interest

The authors declare that the research was conducted in the absence of any commercial or financial relationships that could be construed as a potential conflict of interest.

## Publisher’s note

All claims expressed in this article are solely those of the authors and do not necessarily represent those of their affiliated organizations, or those of the publisher, the editors and the reviewers. Any product that may be evaluated in this article, or claim that may be made by its manufacturer, is not guaranteed or endorsed by the publisher.
